# Agonist-Directed Desensitization of the β_2_-Adrenergic Receptor

**DOI:** 10.1371/journal.pone.0019282

**Published:** 2011-04-26

**Authors:** Vasiliy Goral, Yan Jin, Haiyan Sun, Ann M. Ferrie, Qi Wu, Ye Fang

**Affiliations:** Biochemical Technologies, Science and Technology Division, Corning Inc., Corning, New York, United States of America; Cornell University, United States of America

## Abstract

The β_2_-adrenergic receptor (β_2_AR) agonists with reduced tachyphylaxis may offer new therapeutic agents with improved tolerance profile. However, receptor desensitization assays are often inferred at the single signaling molecule level, thus ligand-directed desensitization is poorly understood. Here we report a label-free biosensor whole cell assay with microfluidics to determine ligand-directed desensitization of the β_2_AR. Together with mechanistic deconvolution using small molecule inhibitors, the receptor desensitization and resensitization patterns under the short-term agonist exposure manifested the long-acting agonism of salmeterol, and differentiated the mechanisms of agonist-directed desensitization between a full agonist epinephrine and a partial agonist pindolol. This study reveals the cellular mechanisms of agonist-selective β_2_AR desensitization at the whole cell level.

## Introduction

The β_2_-adrenergic receptor (β_2_AR) is involved in controlling smooth muscle relaxation in the airways and the vasculature, and in regulating many other physiologically important processes [1]–[4]. β_2_AR selective agonists are the first-line medications for relief of life-threatening bronchospasm, the hallmark feature of asthma and chronic obstructive pulmonary disease. However, prolonged or repeated use of current β_2_-agonist drugs leads to loss of their effects, a pervasive phenomenon termed tachyphylaxis, refractoriness, or desensitization [5], [6]. β_2_-agonists with reduced tachyphylaxis are postulated to lead to improved tolerance profiles, thus therapeutically advantageous [7]. Molecular pharmacology assays have advanced our understanding in the molecular mechanisms of receptor desensitization and resensitization [7], [8]. However, comprehension about how receptor desensitizes and resensitizes at the systems level still lags behind, mostly due to lack of assays that not only effectively detect cell signaling at the whole cell or pathway level, but also enable mechanistic deconvolution. The molecular assays often have low temporal resolution, and receptor desensitization is mostly inferred at the single signaling molecule level. Physiological assays do describe receptor desensitization at the systems level, but the resultant hemodynamic parameters are too complex to derive mechanistic descriptions of receptor desensitization.

Resonant waveguide grating (RWG) biosensor is a label-free optical biosensor that has been shown to be able to non-invasively measure receptor signaling and ligand pharmacology in live cells [9]–[12]. The resultant signal, termed as dynamic mass distribution (DMR), is an integrated and whole cell response that reflects signaling pathway(s) involved downstream the receptor activation [13]–[15]. However, in typical biosensor cellular assays a ligand is introduced once using a pipetting system and cells are continuously exposed to the ligand, thus creating a static and sustained stimulation scheme [16]. Such an assay format may mask certain biased activities of various ligands. Given the pleiotropic pathways of β_2_AR signaling [8] and the ability of distinct agonists to stabilize different active conformations of the receptor [17]–[19], it is postulated that distinct agonist-activated conformations could result in different molecular mechanisms of receptor desensitization [20]. Herein, using the β_2_AR in A431 cells as a model, we attempt to elucidate the mechanisms of β_2_AR desensitization and resensitization at the whole cell level, and to characterize the agonist-selective desensitization of the β_2_AR using a microfluidic biosensor system.

## Results

### Characteristics of microfluidic biosensor system

**Figure 1:**
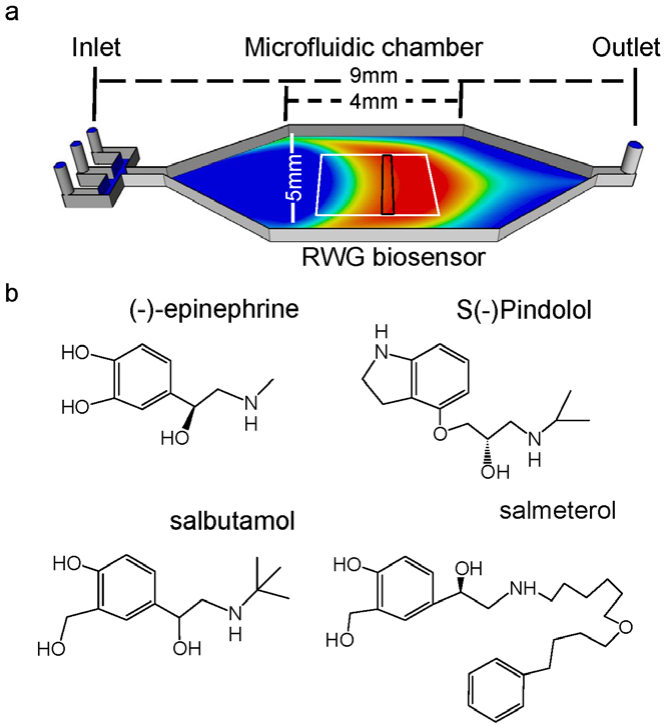
A microfluidic RWG biosensor array for characterizing ligand-directed desensitization of the β_2_-adrenergic receptor. (a) Schematic of the biosensor microchamber, wherein an agonist solution, 2 µl in total, is perfused at a flow rate of 1 µl/min between two perfusion steps with the assay buffer, thus creating 2 min pulse stimulation to the cells located within the detection area (black box). During the time the agonist solution passing through the microchamber, small diffusion occurs. Since the detection area (0.2 mm) is smaller than the width of the agonist solution (∼2 mm for the 2 min pulse stimulation), the agonist concentration exposed to the cells within the detection area is considered to be constant. The white box indicates the location of the biosensor. (b) Structures of four β_2_-agonists examined.

The microfluidic biosensor system contains three key components: a 4×4 array of functional RWG biosensors, a microfluidic device, and a label-free imager. The biosensor array was made onto a glass substrate using a microfabrication process developed in house [21], each biosensor having a dimension of 2 mm×2 mm and the array having a footprint that is compatible to a standard 384-well microplate. The microfluidic device contains a 4×4 array of microfluidic chambers, each having three inlets and one outlet, and the microchamber array covering 4×8 array of biosensors so only one biosensor is centered in each microchamber and the adjacent biosensor is sacrificed. After assembled onto the substrate, syringe pumps were connected to the inlets to deliver solutions at a controlled flow rate. The distance from an inlet to the outlet is 9 mm, the central width of the chamber 5 mm, and the height of the microchannel 200 µm (Fig. 1a). The total volume required to fill up a chamber is 6 µl.

The imager is based on a swept wavelength interrogation detection scheme, which uses an expanded beam from a tunable laser light source to simultaneously illuminate at normal incident angle the biosensor array [22]. This imager has a spatial resolution of 25 µm and a temporal resolution of 3 sec.

To estimate the shear stress inside the microfluidic chamber we approximated the flow profile in the middle of the chambers by parallel-plate Poiseuille flow [23]. Thus, the fluidic shear on the cells can be modeled by assuming that it would be equal the shear stress σ at the wall between parallel plates under parabolic flow [22]:
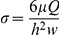
where *μ* is the fluid viscosity, *Q* the fluid flow rate, *h* the chamber height, and *w* the chamber width. This does not account for changes in flow due to the presence of the adherent cells, but it should be a reasonable approximation because *w/h* is large [25] and cell layer is only 2–4 µm high. Considering the current configurations wherein *Q* is 1 µl/min, *μ*≈10^−3^ Pa·s, *h* = 0.2 mm, and *w* = 5 mm, we estimated that a wall shear stress σ is about 5×10^−4^ Pa, or a shear force is about 5×10^−3^ dyn/cm^2^. Such a low shear force is well below physiological values of 0.5–2 Pa, as well as those commonly used to cause the activation of selective signaling cascades such as extracellular signal-regulated kinases (ERKs) and N-terminal jun kinase in certain cells [24]–[27].

### Potency of the β_2_AR agonists to trigger DMR signals in A431 cells

Based on known agonism activity and lipophilicity, four β_2_-agonists were chosen to characterize the ligand-selective desensitization in A431, a human epidermoid carcinoma cell line that endogenously expresses β_2_AR, but not other adrenergic receptors [28]. Both epinephrine and salbutamol are hydrophilic agonists, while pindolol is a moderately lipophilic partial agonist, and salmeterol is a lipophilic long-acting agonist (Fig. 1b). DMR assays under the static stimulation condition suggest that a single EC_50_ was obtained for epinephrine (0.07±0.03 nM), S(-)pindolol (0.04±0.02 nM), and salbutamol (0.90±0.15 nM) (n = 3). However, salmeterol led to a biphasic dose response with two separated EC_50_ (0.2±0.04 nM and 155±23 nM, respectively) (n = 3). These results were consistent with our previous study [29]. Thus we chose the 1× EC_100_ dose for each agonist to study receptor desensitization. The dose was 2 nM, 5 nM, 10 nM, and 100 nM, for epinephrine, pindolol, salbutamol, and salmeterol, respectively.

### Epinephrine stimulation duration dependent signaling of the β_2_AR

**Figure 2:**
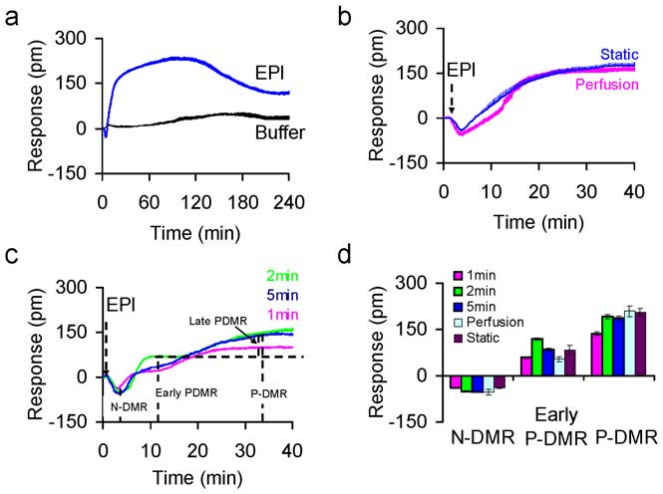
The epinephrine DMR in quiescent A431 cells is sensitive to stimulation duration. (a) static and sustained stimulation within 4 hrs, the buffer induced response was included as a negative control; (b) sustained stimulation with perfusion, in comparison with static and sustained stimulation within 40 min; (c) pulse stimulation conditions (1 min, 2 min, and 5 min) followed by continuous perfusion with the assay buffer. (d) The amplitudes of N-DMR, early P-DMR (8 min post stimulation) and the P-DMR in total (30 min post stimulation) for the epinephrine DMR as a function of stimulation conditions. Epinephrine dose was 2 nM for all. The cells were monolayer under all conditions. The standard deviation represents at least 3 replicates in measurements.

Under the static stimulation condition epinephrine of 2 nM induced a DMR that consists of a rapid negative-DMR (N-DMR), a succeeding positive-DMR (P-DMR) leading to a stably elevated level for ∼2 hrs, followed by a slowly decayed DMR to another elevated plateau (Fig. 2a). The sustained stimulation with epinephrine under the shear flow led to almost identical DMR (Fig. 2b). However, under pulse stimulation conditions epinephrine resulted in clear agonist exposure time dependent DMR signals (Fig. 2b and c), but whose persistences were similar to those under static stimulation condition. Interestingly, the pulse stimulation split the single P-DMR into a biphasic event: a rapid early P-DMR followed by a late P-DMR. Such an alteration in DMR characteristics suggests that the activation of the β_2_AR by epinephrine leads to multiple pathways and certain cell signaling events require persistent agonist occupancy. Furthermore, results also showed that regardless of exposure time the epinephrine DMR remained at the elevated level for at least 2 hrs after agonist removal, suggesting that once the β_2_AR signaling propagates, it remains committing to regulate the whole cell response.

**Figure 3:**
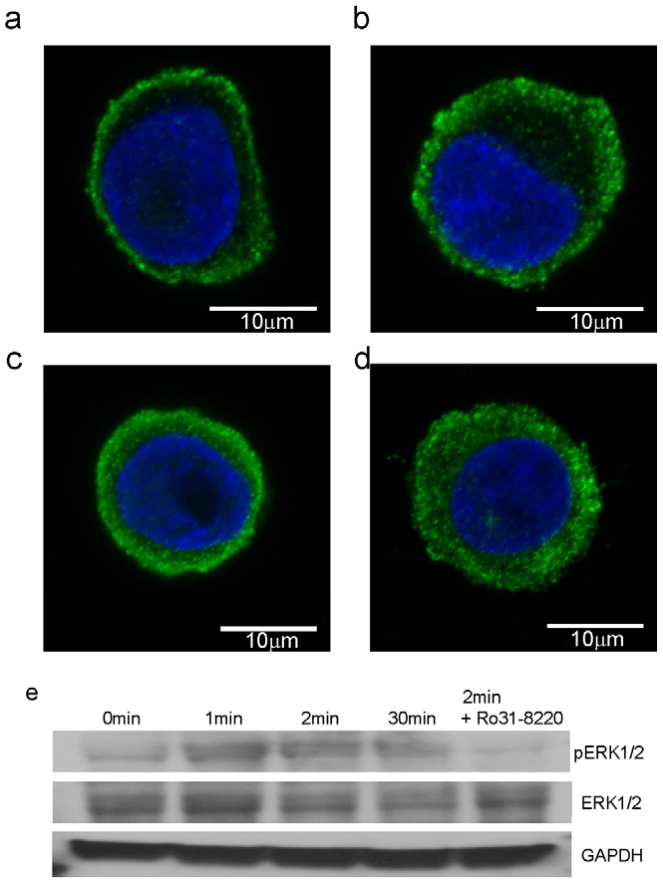
Receptor internalization and ERK1/2 phosphorylation are dependent on the agonist exposure time. (a)–(d) Confocal images of cells after immunostained with anti-β_2_AR. The cells were first stimulated with 2 nM epinephrine for 1 min (a), 2 min (b), 5 min (c) and 30 min (d). After epinephrine removal, the cells were further incubated in medium at 37°C for 29 min (a), 28 min (b), 25 min (c) and 0 min (d), respectively, such that equal time post stimulation was achieved for all. The staining was performed using anti-β_2_AR antibody and Alexa Fluor 488 goat anti-rabbit antibody. The nuclei were stained with DAPI. Confocal microscopy images were acquired on Zeiss confocal laser scanning microscope (oil-immersion, 63× objective). The green color is from the β_2_AR, the blue from the nuclei. (e) The agonist exposure time and Ro31-8220 sensitivity of pERK stimulated by epinephrine in A431 cells. The cells were incubated with the vehicle (0 min) or 2 nM epinephrine for a specific time (1 min, 2 min, 30 min), followed by further incubation at 37°C for another time to ensure all reach 30 min post simulation. Ro31-8220 when used was presented throughout the incubation. Equal amounts of cell lysate were separated by SDS-PAGE and analyzed for pERK by Western blotting. Equivalent gel loading was confirmed by probing with antibodies against GAPDH.

Since the epinephrine DMR was found to be sensitive to the agonist exposure time, the degrees of receptor internalization under different stimulation schemes were examined. Considering the slow kinetics of receptor internalization, all cells were examined 30 min later since the epinephrine exposure started. Results showed that similar to unstimulated cells, the β_2_AR mostly remained at the cell surface in the 1 min pulse stimulated cells (Fig. 3a). The 2 min pulse stimulation caused small number of receptors internalized (Fig. 3b). Receptor internalization became obvious in the 5 min pulse stimulated cells (Fig. 3c), and mostly completed in the cells treated continuously with epinephrine for 30 min (Fig. 3d). Thus, this is a clear negative correlation between the receptor internalization and the occurrence of the early P-DMR event. This is consistent with our previous finding that β_2_AR internalization is a negative contributor to the epinephrine P-DMR event [29].

Since ERK1/2 phosphorylation is a hallmark signature of the β_2_AR activation, we were interested in the impacts of agonist exposure time on ERK1/2 phosphorylation. Similar to internalization, pERK level was measured 30 min later since the stimulation started regardless of agonist exposure time. Immunoblotting showed that all stimulations resulted in moderate increase in pERK, but the pulse stimulations led to more sustained pERK (Fig. 3e). Furthermore, the co-incubation with an ERK inhibitor, Ro31-8220, blocked the epinephrine-induced ERK activation. Taken together, these results suggest that the β_2_AR signaling is sensitive to the duration of epinephrine occupancy, and certain pathways downstream the receptor is persistent after epinephrine removal.

### Epinephrine induced desensitization and resensitization of the β_2_AR signaling under different stimulation conditions

**Figure 4:**
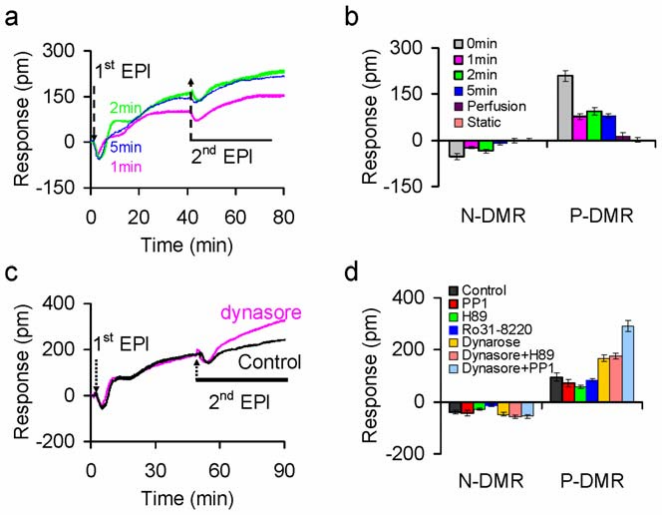
The desensitization and resensitization patterns of quiescent A431 cells induced by epinephrine is sensitive to stimulation duration and several inhibitors. (a) The DMR signals upon repeated stimulations with epinephrine. After initial baseline (∼2 min), the cells were first stimulated with epinephrine (1^st^ EPI) for three different durations (1 min, 2 min, or 5 min), followed by perfusion with the assay vehicle for ∼35 min, and finally stimulated again with a continuous flow of epinephrine (2^nd^ EPI) for ∼30 min. (b) The amplitudes of both N-DMR and P-DMR of the 2^nd^ EPI induced DMR as a function of the 1^st^ stimulation conditions. (c) The epinephrine DMR in the absence and presence of dynasore. The cells were first stimulated with epinephrine for 2 min (1^st^ EPI) in the presence and absence of dynasore, followed by perfusion with the assay buffer in the absence and presence of dynasore, respectively. Afterwards, all the cells were repeatedly stimulated with epinephrine in the absence of dynasore (2^nd^ EPI). (d) The amplitudes of both N-DMR and P-DMR of the 2^nd^ EPI induced DMR as a function of inhibitors. Each inhibitor or their combinations were assayed in a manner similar to dynasore. The flow rate was 1 µl/min under all conditions. Inhibitor concentrations were 5 µM, 10 µM, 10 µM, and 50 µM for H-89, Ro31-8220, PP1, and dynasore, respectively. Epinephrine dose was 2 nM for all. n = 3.

Desensitization and resensitization of the β_2_AR signaling caused by epinephrine was then examined under different exposure times. Results showed that the desensitization of the β_2_AR signaling is also dependent on the epinephrine exposure time. When cells were exposed to 2 nM epinephrine for a short period of time (1 min, 2 min or 5 min), the cells still responded to the second epinephrine stimulation but with a suppressed DMR signal (Fig. 4a), suggesting that epinephrine removal caused resensitization of the β_2_AR signaling. Conversely, under the two sustained stimulation conditions (*i.e.*, static and continuous flow) the cells completely lost their responsiveness to the second stimulation (Fig. 4b). These results suggest that the β_2_AR signaling at the whole cell level is desensitized even when the receptor occupancy by epinephrine is low.

To examine the cellular mechanisms of β_2_AR desensitization and resensitization, several small molecule inhibitors were used. Results showed that these inhibitors impact differently the DMR pattern to repeated epinephrine stimulations (Fig. 4c and Fig. 4d). For the 2^nd^ epinephrine stimulation induced DMR, the DMR remained unchanged by PP1 and Ro31-8220, but was slightly suppressed by H-89. The most notable is that dynasore potentiated the DMR, while the co-presence of dynasore with PP1, but not H-89, further increased the DMR. PP1 is a known Src inhibitor, H-89 is a PKA inhibitor, and dynasore is a dynamin inhibitor. These results suggest that dynamin- and Src-regulated processes are important to the epinephrine mediated desensitization and resensitization of the β_2_AR.

### Agonist-directed desensitization of the β_2_AR signaling at the whole cell level

**Figure 5:**
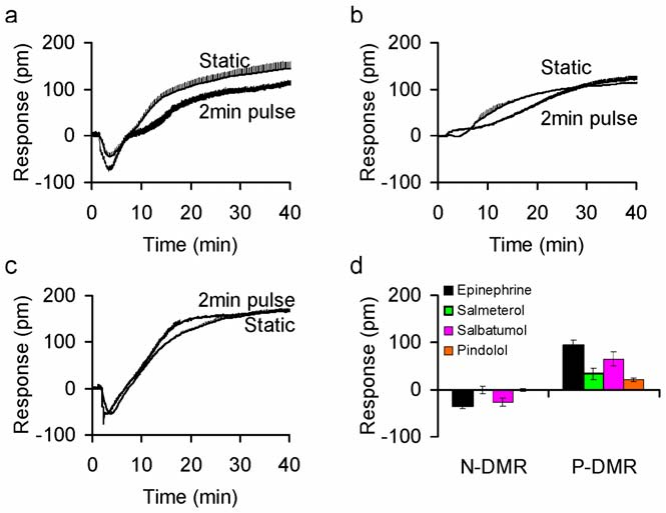
The DMR signals induced by distinct β_2_AR agonists under distinct conditions. (a) salbutamol, (b) pindolol, and (c) salmeterol, under the 2 min pulse stimulation in comparison with the sustained stimulation conditions. (d) The amplitudes of both N-DMR and P-DMR (30 min post stimulation) of the 2^nd^ epinephrine stimulation induced DMR as a function of different agonists. For receptor desensitization studies, two steps were separated by ∼45 min. The initial stimulation duration with an agonist was 2 min, while the second stimulation was continuous. The concentration was 2 nM, 5 nM, 10 nM, and 100 nM for epinephrine, pindolol, salbutamol, and salmeterol, respectively. n = 3.

Next, agonist-selective desensitization was examined. The pulse stimulation duration was set to be 2 min for all. Results showed that salbutamol behaved similarly to epinephrine, both resulting in a separation of the single phase P-DMR under the sustained stimulation into the biphasic P-DMR events (Fig. 5a). Pindolol led to a DMR similar to that obtained under the sustained stimulation, but with a slower kinetics (Fig. 5b). Conversely, salmeterol led to a DMR identical to that obtained under the sustained stimulation (Fig. 5c). Furthermore, the pulse stimulation also caused an agonist-dependent receptor desensitization and resensitization pattern (Fig. 5d). Under the pulse stimulation condition, both salmeterol and pindolol caused complete desensitization to repeated epinephrine stimulation, while epinephrine and salbutamol caused partial desensitization. The resensitization degree is higher for epinephrine than salbutamol. Thus, these results suggest that the microfluidic biosensor differentiates ligand-directed desensitization of the β_2_AR.

**Figure 6:**
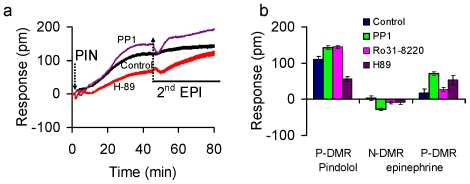
The DMR patterns of cells upon repeated stimulation with pindolol and epinephrine. Step 1: 2 min pulse stimulation in the absence and presence of an inhibitor, followed by perfusion with the assay buffer in the absence and presence of the respective inhibitor; and Step 2: continuous exposure to 2 nM epinephrine using perfusion. (a) DMR signals, and (b) DMR characteristics, later of which the P-DMR of the 1^st^ pindolol-induced DMR, and both N-DMR and P-DMR of the 2^nd^ EPI induced DMR were plotted as a function of inhibitors. The flow rate was 1 µl/min under all conditions. n = 2 to 4.

The desensitization mechanism caused by pindolol was further examined, since it was unexpected that pindolol behaved similarly to salmeterol, both of which caused complete desensitization to the repeated stimulation with epinephrine. Results showed that the pindolol DMR itself was found to be potentiated by PP1 and Ro31-8220, but suppressed by H-89 (Fig. 6a). However, the 2^nd^ epinephrine stimulation induced DMR displayed different patterns – the epinephrine DMR was the greatest in the presence of PP1, followed by H-89>Ro31-8220 (Fig. 6b). These results suggest that both Src and PKA are important in regulating the desensitization caused by pindolol.

**Figure 7:**
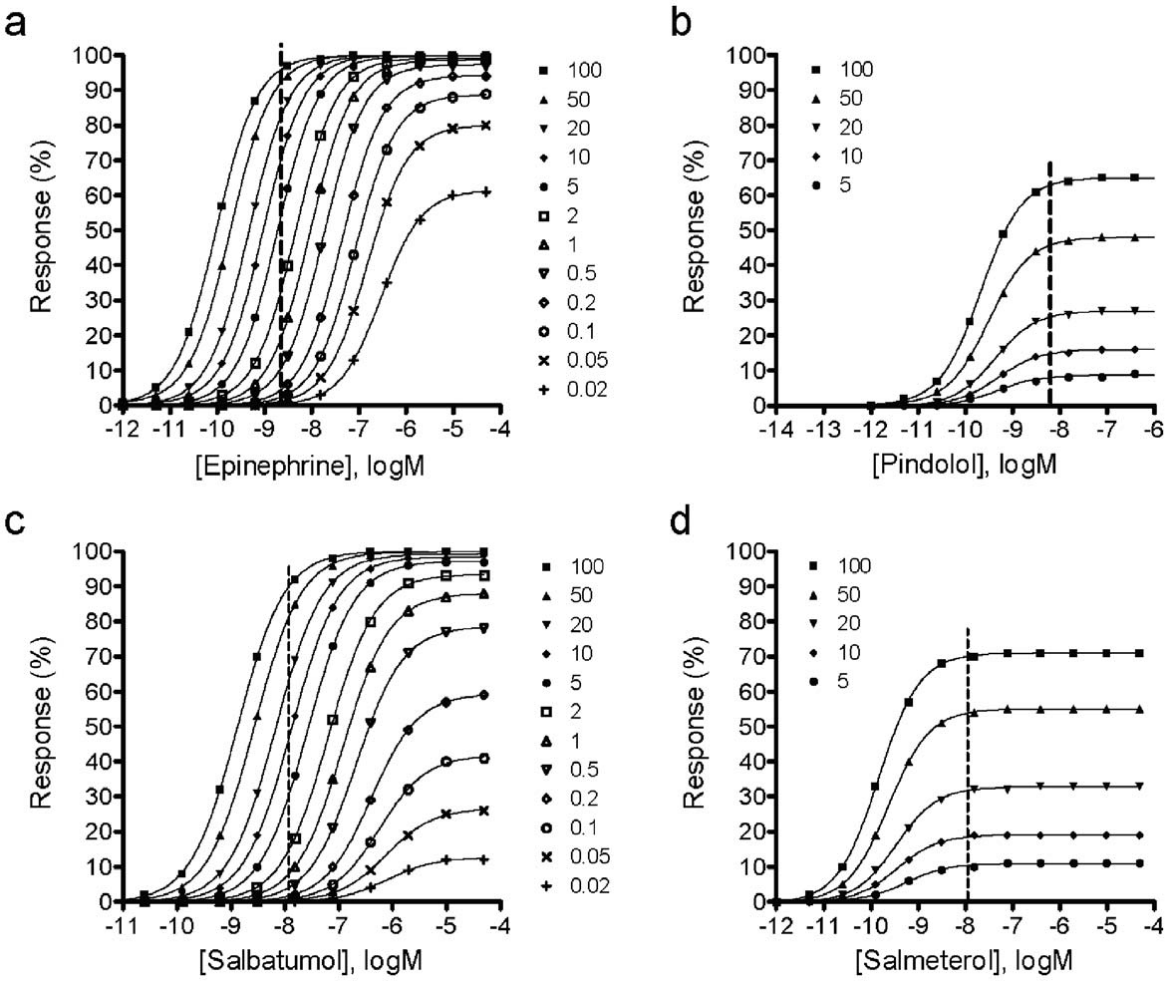
The simulated dose responses of distinct agonists as a function of spare receptors in the cell using the operational model. (a) epinephrine; (b) pindolol; (c) salbutamol; and (d) salmeterol. The spare receptors were normalized in percentage.

**Figure 8:**
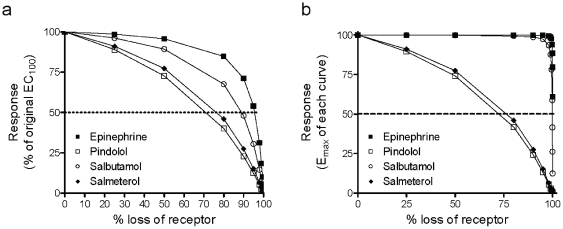
The simulated sensitivity of distinct agonists to loss of receptors (in percentage). (a) The effect of an EC_100_ of each agonist (calculated where the total receptor is unchanged; *R_T_* = 100) at different receptor numbers. Data were normalized to percentage of the response at the EC_100_ where *R_T_* = 100. (d) The function of *E_max_* achievable by the agonists as receptor loss.

To understand the ligand-selective receptor desensitization, we determined relative efficacy of the β_2_AR agonists in DMR assays using the Black and Leff model of operational agonism [30]. It defines agonist [A] response as [31]

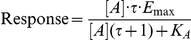
Where [A] is the agonist concentration, *E_max_* the maximal response of A431 cell, *K_A_* the equilibrium dissociation constant of the ligand, and *τ* an efficacy term equal to the ratio of receptor density (*R_T_*) to *K_e_*, where *K_e_* is the efficiency of signal transduction by the ligand-receptor complex. The K_A_ values of epinephrine, pindolol, salbutamol and salmeterol were obtained from literature, and were 741.3, 0.6, 977.2, and 0.5 nM, respectively [32]. Fitting the dose-dependent DMR response of each ligand with the operational mode led to a *τ* value of 7904±678, 1.86±0.10, 721±94, and 2.42±0.14 for epinephrine, pindolol, salbutamol and salmeterol, respectively. The dose response as a function of receptor level was then simulated for each agonist using the operational model (Fig. 7). The reduced spare receptors were used to mimic desensitization. Results showed that as expected the apparent potency and efficacy of all agonists decreased as the total receptors decreased, but different agonists exhibited distinct sensitivity to the total receptor numbers. Both epinephrine and salbutamol exhibited higher efficacy and were less sensitive to loss of receptor than pindolol and salmeterol. Further, the responses of the β_2_AR agonists at their original EC_100_ showed distinct sensitivity to loss of receptor, with the order of pindolol>salmeterol>salbutamol>epinephrine (Fig. 8a), while the maximal achievable responses by pindolol and salmeterol were more sensitive to loss of receptors than those by epinephrine and salbutamol (Fig. 8b). This is consistent with our results obtained under the pulse stimulation condition, when the repeated stimulation with epinephrine of 2 nM was used as readout of receptor desensitization and resensitization (Fig. 5d).

## Discussion

The present method is greatly different from conventional receptor desensitization and resensitization studies. First, receptor desensitization and resensitization was carried out at the whole cell level enabled by label-free DMR assays, instead of single signaling molecule(s) downstream the receptor activation. Second, as a step closer to clinical settings where doses given to patients are based on functional effect but not receptor occupancy [33], [34], we chose equi-effective doses, instead of doses to achieve equal receptor occupancy, for the desensitization studies. Third, since the receptor activation by endogenous agonists often occur under pulse stimulation conditions (e.g., synaptic transmission) [35], we chose a pulse stimulation scheme to achieve receptor desensitization and a short duration of receptor recovery to study receptor resensitization. Fourth, the non-invasive and real time kinetic measures of DMR assays not only allow us track the process of signaling propagation when stimulation is terminated, but also make it possible to deconvolute the pathway biased mechanisms of distinct agonists to cause receptor desensitization.

The RWG biosensor non-invasively monitors a cellular response and converts it into a biosensor signal, termed DMR. The DMR is reported as a shift in resonant wavelength (in picometer). Since the biosensor is sensitive to alterations in local refractive index which is directly correlated to the density of local biomaterials (e.g., proteins), DMR defines the redistribution in local biomass within the sensing volume of the biosensor [15]. Combining with chemical biology and conventional cell biology approaches, we and others have found that DMR faithfully reflects the early cell signaling pathways downstream of receptor activation [11]. In our recent and present studies we have found that for the epinephrine-induced DMR of the β_2_AR in A431, (1) it is largely downstream of adenylate cyclase activation [36]; (2) it involve multiple pathways including Src, PKA-MAPK, and dynamin [36]; (3) the receptor internalization is a negative contributor to its P-DMR event in which receptor internalization slows down its kinetics [29]; (4) the actin remodeling, but to less extent microtubule remodeling, contributes to its early DMR event [30]; and (5) other cellular events including the β_2_AR activation induced increase in cell adhesion may also contribute to its DMR.

To study ligand-directed desensitization of the β_2_AR, we developed a microfluidic biosensor system. The system enables precise controls of agonist exposure time and the duration of the functional recovery of activated receptors with a moderate throughput (16 independent measurements in parallel). DMR assays under different conditions revealed two types of β_2_AR agonists. Comparing to their respective DMR signals obtained under two (static and perfusion) sustained stimulation conditions, the pulse stimulation resulted in more obvious alterations in characteristics of DMR signals induced by epinephrine and salbutamol, compared to pindolol or salmeterol. Such different behaviors appear correlated well with the difference in lipophilicity of distinct agonists. Unlike epinephrine and salbutamol both of which are hydrophilic and short-acting agonists, both salmeterol and pindolol are lipophilic, thus leading to long-acting agonism.

The biosensor cellular assays with microfluidics allow ligand-directed desensitization to be studied at the whole cell level, instead of measurements based on single signaling molecules using conventional assays. Results showed that at the whole cell level the β_2_AR signaling is desensitized even at low receptor occupancy by agonists of high intrinsic efficacy such as epinephrine or salbutamol. Further, the desensitization and resensitization pattern was found to depend on the types of agonists and stimulation conditions. Both pindolol and salmeterol led to complete desensitization to the repeated stimulation with epinephrine, even when the initial stimulation duration was short as 2 min. However, both epinephrine and salbutamol resulted in desensitization patterns that are dependent on the initial agonist exposure duration. The shorter the initial stimulation the greater resensitization is. Using small molecule inhibitors, the biosensor cellular assays with microfluidics also enable mechanistic deconvolution of signaling pathways that lead to receptor desensitization. Results showed that the epinephrine pulse stimulation induced desensitization was sensitive to both Src and dynamin, while the pindolol pulse stimulation induced desensitization was related to Src and PKA. Together, these results suggest that the β_2_AR desensitization is ligand-selective.

In conclusion, the label-free and microfluidic RWG biosensor enables precise control the times of both agonist exposure and functional recovery of the agonist-activated receptors. Using the endogenous β_2_AR in A431 as a model, ligand-directed desensitization of the β_2_AR was manifested – both salmeterol and pindolol exhibit long-acting agonism, when epinephrine and salbutamol display short acting agonism. Furthermore, epinephrine caused receptor desensitization at the whole cell level via the dynamin and Src-mediated processes, while pindolol caused receptor desensitization mostly via PKA and Src. Scaling up the biosensor system will open new means to screen novel β_2_-agonists with reduced tachyphylaxis.

## Materials and Methods

### Materials

Epinephrine, salmeterol, pindolol and salbutamol were obtained from Sigma Chemical Co. (St. Louis, MO, USA). PP1, H-89, Ro31-8220 and dynarose were obtained from Tocris (St. Louis, MO, USA). Rabbit polyclonal anti-β_2_AR (#sc-569), mouse monoclonal anti-pERK1/2 (#sc-81492) and rabbit polyclonal anti-GAPDH (#sc-25778) antibodies were purchased from Santa Cruz Biotechnology Inc. (Santa Cruz, CA, USA). Mouse monoclonal anti-ERK1/2 (#L38C12) was obtained from Cell Signaling Technology Inc (Danvers, MA). Alexa Fluor 488 goat anti-rabbit antibody was purchased from Invitrogen (Carlsbad, CA, USA). Epic® 384-well biosensor microplates and inserts were obtained from Corning Inc. (Corning, NY, USA). Poly-dimethylsiloxane (PDMS) was obtained from Dow Chemical (Midland, MI, USA).

### Fabrication of microfluidic biosensor devices

Soft lithography was used to fabricate the microfluidic biosensor device using PDMS replicas [37]. Briefly, features on chrome mask were transferred onto silicon wafers using standard photolithographic process. The photoresist-defined silicon wafers were then anisotropically etched to a desired depth in a multiplex inductively coupled plasma etching system (Surface Technology Systems, Newport, NJ, USA). After photoresist removal and overnight coating using trichloro(1H,1H,2H,2H-perfluorooctyl)silane, a PDMS pre-polymer solution containing a mixture (10∶1 mass ratio) of PDMS oligomers and a reticular agent (Sylgard 184® Kit, Dow Corning Corp., Midland, MI, USA) was cast onto the etched silicon wafers and cured at room temperature for about 24 hrs to minimize shrinkage after curing. Next, the PDMS replicas were carefully peeled away from the silicon wafers. Finally, after punching inlet and outlet holes, the PDMS replica was aligned and reversibly bonded onto the top of biosensor inserts.

### Cell culture

Human epidermoid carcinoma A431 cells were obtained from American Type Cell Culture. The cells were grown in the cell culture medium (Dulbecco's modified Eagle's medium (DMEM) supplemented with 10% fetal bovine serum (FBS), 4.5 g/liter glucose, 2 mM glutamine, and antibiotics). The native cells were passed when approaching ∼90% confluence with trypin/EDTA (etthylenediaminetetraacetic acid) to provide new maintenance cultures on T-75 flasks and experimental cultures on the biosensor microplates or chambers. The cells up to at least 20 passages are appropriate for the biosensor cellular assays in our laboratory.

For cell culture within the microfluidic biosensor device, the device, tubing (Tygon S-54-HL, Saint-Gobain Performance Plastics, Akron, OH, USA) and syringes (500 µl, gas tight 1700 series, Hamilton, Reno, NV, USA) were sanitized with 70% ethanol. After sanitization, the microfluidic chambers were filled with the cell culture media. Cells after harvested using tyrpsinization were freshly suspended in the medium. 4×10^4^ cells suspended in 6 µl of the medium were injected into each chamber. Cells were allowed to seed via 30 min incubation at room temperature. Tubing was then plugged into the microchamber inlets and was connected to syringes that were connected to a syringe pump (Model: SP230IW; World Precision Instruments, Sarasota, FL, USA). To prevent any evaporation, the biosensor device was maintained within a petri dish with a cover, and extra cell medium within the dish. Next, the cells were cultured at 37°C under air/5% CO_2_ and perfusion of the serum rich media was started at a flow rate of 5 µl/hr. After overnight culturing, the cells reached a confluency of about 95%. The cells were then subject to overnight starvation with the medium without any FBS.

For cell culture under regular Epic biosensor microplates, 20000 cells per well, suspended in the DMEM medium containing 10% FBS, were directly seeded into the microplates. After overnight culturing, the cells were subject to 24 hrs starvation using the serum-free medium.

### DMR assays under static stimulation conditions using Epic® system

Epic® system (Corning Inc., Corning, NY, USA) is a wavelength interrogation reader system tailored for resonant waveguide grating (RWG) biosensors in microtiter plates, and used for DMR assays under static stimulation conditions [21]. This system consists of a temperature-control unit, an optical detection unit, and an on-board liquid handling unit with robotics. The detection unit is centered on integrated fiber optics, and enables kinetic measures of cellular responses with a time interval of ∼15 sec. After culturing, the cells were washed with the assay buffer (1× HBSS, Hanks' balanced salt solution, 20 mM Hepes, pH 7.1) and incubated within the system for about 1 hr. Afterwards a 2-min baseline was established. Compound solutions were then transferred into the biosensor wells using the on-board liquid handling device, and the cell responses were recorded in real time. During the assay, the compounds added remain inside the wells. All studies were carried out with at least three replicates, unless specifically mentioned.

### DMR assays under microfluidic environments using a RWG imager system

A whole microplate RWG biosensor imager system was recently developed [22]. This system was further modified to increase spatial resolution as well as to accommodate the reduced footprint of the current microfluidic biosensor devices. Specifically, a tunable light source is passed through a polarizer to generate a polarized light, which is then expanded through optical lens and mirrors to simultaneously illuminate at a normal incident angle all biosensors within a microfluidic device. The tunable light source linearly scans the wavelength range from 826 nm to 838 nm in every 3 seconds, such that the biosensor array is illuminated and synchronously imaged by a 1400×1024 pixel complementary metal oxide semiconductor camera (Dalsa Inc., Ontario, Canada). The spectral images are processed online to extract the resonant wavelengths (in picometer, pm) and their changes over time. In order to minimize assay variability as well as the impact of time lag across the biosensor, cells only located within the central area of a biosensor (0.2×2 mm) were sampled to generate an averaged response. For DMR assays under microfluidics, microchambers with confluent layer of cells after culturing were connected to three independently operated syringes using Tygon Ò tubing. Initially cells were perfused with the assay buffer at a flow rate of 1 µl/min until a baseline signal was established and normalized to zero. By switching operational pumps cells were then stimulated with a ligand for a specific period of time, followed by perfusion with the assay buffer for 40 min to allow functional recovery of activated receptors. Finally the cells were stimulated again with a continuous flow of epinephrine solution for 30 min. The three independently operated inlets allowed us to perform continuous perfusion of cells during the assay without any abrupt changes of shear stress or laminar flow perturbation inside the microfluidic chamber.

### Receptor internalization assays

A431 cells were grown on glass chamber slides (Nunc, Rochester, NY). After treatment with epinephrine for a given period, the agonist solution was replaced with the serum free medium and further incubated for another specified period at 37°C inside a standard cell culture incubator. The cells were then fixed with 2% formaldehyde in phosphate buffered saline solution (PBS), and permeabilized with 0.1% Triton X-100 in PBS. After blocking using bovine serum at room temperature, cells were incubated overnight with primary antibody (anti-β_2_AR antibody) at 4°C. This was followed by three washing steps with PBS, an 1 hr incubation with Alexa Fluor 488 goat anti-rabbit antibody, and final washing steps. Then, samples were imbedded in mounting medium containing DAPI, a nuclei staining agent (Vector Labs, Burlingame, CA). Confocal microscopy images were acquired on ZEISS confocal Laser Scanning Microscope (oil-immersion, 63× objective). The digital images were processed with ImageJ (http://rsbweb.nih.gov/ij/).

### ERK MAPK Assay

The p44/42 MAP kinases were examined using Western blotting. Whole cell lysates were prepared for A431. Cells were either treated or not with epinephrine in the absence and presence of Ro31-8220 for a specified period, followed by further incubation at 37°C in the serum free medium for another specified period. Equivalent gel loading was confirmed by probing with antibodies against GAPDH. The total ERK1/2 and phosphorylated ERK1/2 were blotted using respective antibodies.
